# Maffucci’s Syndrome or a Variant?

**Published:** 2013-05-09

**Authors:** Yousuf Aziz Khan, Soofia Ahmad

**Affiliations:** Department of Paediatric Surgery, National Institute of Child Health, Karachi- 75510, Pakistan

**Keywords:** Maffucci’s syndrome, Enchondromas, Haemangiomas

## Abstract

Maffucci’s syndrome is a rare non hereditary disorder characterized by multiple enchondromas and haemangiomas. A 12 year old boy presented with a painful swelling at his right hand and deformed left upper limb. On detailed workup, he was found to have multiple enchondromas involving long bones and a single haemangioma. A diagnosis of Maffucci’s syndrome was established. The clinical features and workup of the disease in our patient is reported.

## INTRODUCTION

Enchondromas are commonly occurring benign hyaline cartilage neoplasm of medullary bone and are mostly solitary and asymptomatic [1]. Multiple enchondromas (enchondromatosis) in association with soft tissue haemangiomas – Maffucci’s syndrome (MS) was first described by Dr. Angelo Maffucci in 1881 [2]. It is rare and a little more than 200 cases have been reported so far in the world literature with only one case report from our country [3, 4]. The association of enchondromatosis and haemangiomatosis in MS and potential of lesions to undergo malignant degeneration merit detailed workup in such patients. Herein we report a case of a boy with MS and discuss its clinical details.

## CASE REPORT

A 12-year-old boy presented with a painful swelling at his right hand for 7 months. Initially it was barely visible, bluish and non tender, noticed at 6 year of age and gradually increased in size. There was history of trauma to the right hand while playing. He also had history of recurrent swelling left arm after every one to two months with skin eruptions, since birth. It was all treated by local physicians and no medical record was available. There was no complaint of any pain elsewhere in the body. He had excision of a swelling above the right elbow 3 months back but histopathology report was missing. There was no associated significant family history. On examination, a grayish, 5 cm diameter, firm, tender swelling was noted at the mid-palmer aspect of right hand with thick adherent overlying skin, extending dorsally (Fig. 1) There was a pea sized non-tender swelling at the distal phalanx of left index finger and another non-tender swelling at left medial malleolus. His left upper limb was shortened and deformed (Fig. 2). There was a scar mark with a 1 cm swelling above the right elbow. Spleen was enlarged, about 6 cm below the costal margin diagonally. 

**Figure:**
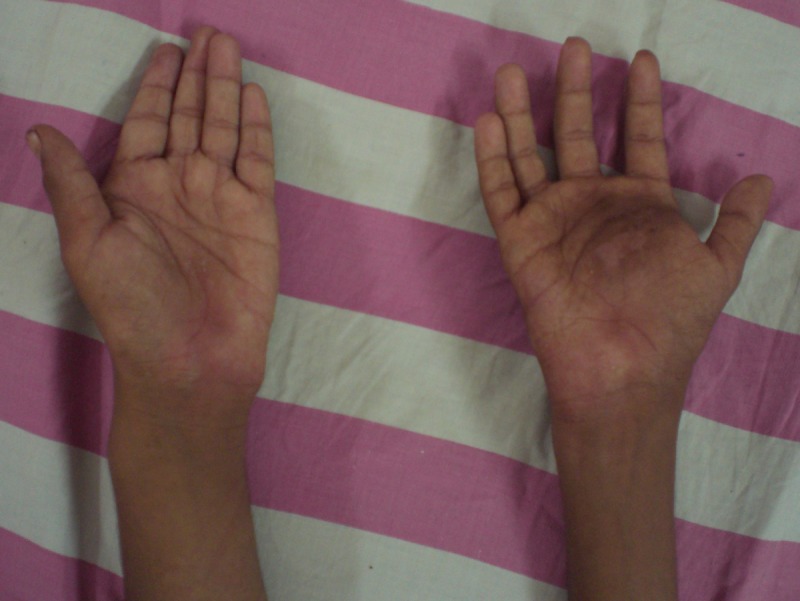
Figure 1: Showing swelling at the right hand. Widening of the left wrist is also noticed.

**Figure:**
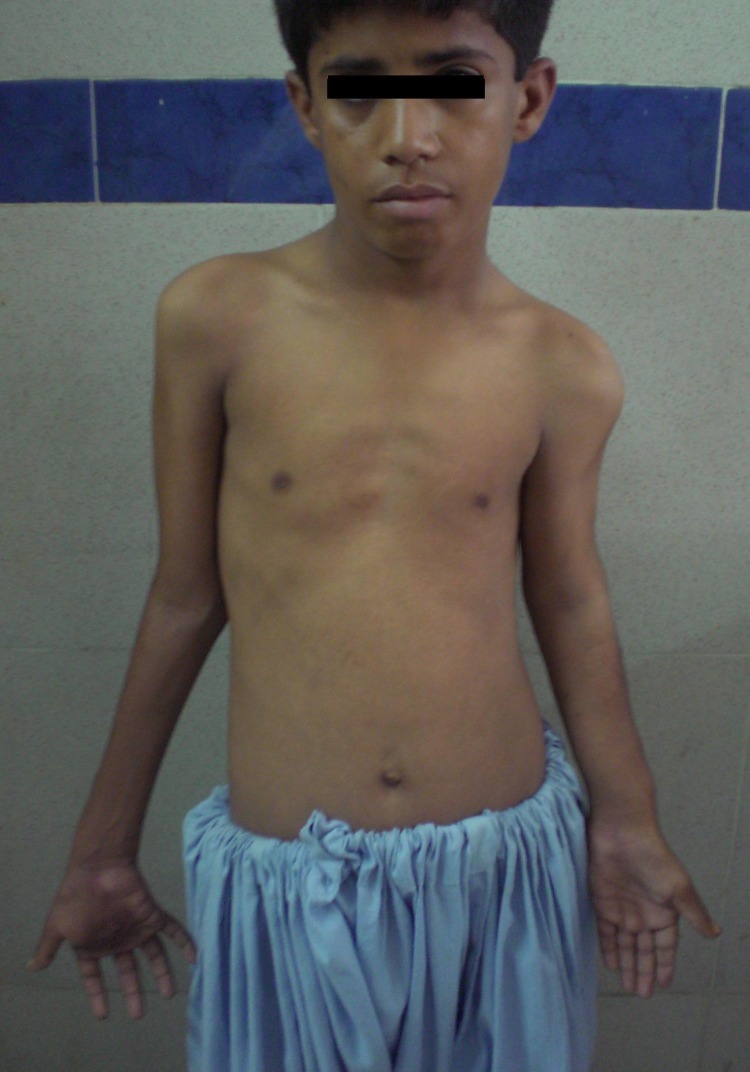
Figure 2: Patient with MS, with deformed, shortened Left upper limb.

Laboratory investigations revealed hemoglobin of 8.7 gm% and alkaline phosphatase levels of 310 U/l. Rest of the reports were within normal limits. Plain radiology revealed soft tissue density swelling with multiple phleboliths involving the right palm. It resulted in widening of the 2nd inter-metacarpal space, scalloping of radial border of 3rd metacarpal and periosteal reaction along the ulnar border of 3rd metacarpal (Fig. 3). Findings were suggestive of haemangioma. Multifocal lytic areas with central areas of calcification were also noticed involving 6th right rib, left scapula, left proximal humerus and distal radius, left proximal and distal fibula, most likely representing enchondromas. No evidence of periosteal reaction or soft tissue extension was noticed (Fig. 4, 5). X-ray-skull and spine were normal. Multifocal enchondromas with soft tissue hemangioma was consistent with Maffucci’s syndrome.

**Figure:**
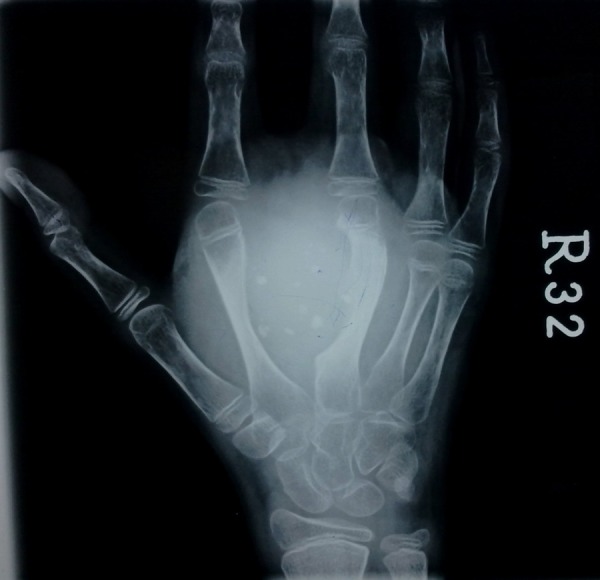
Figure 3: Plain radiograph - right hand: showing soft tissue density swelling with multiple phleboliths.

**Figure:**
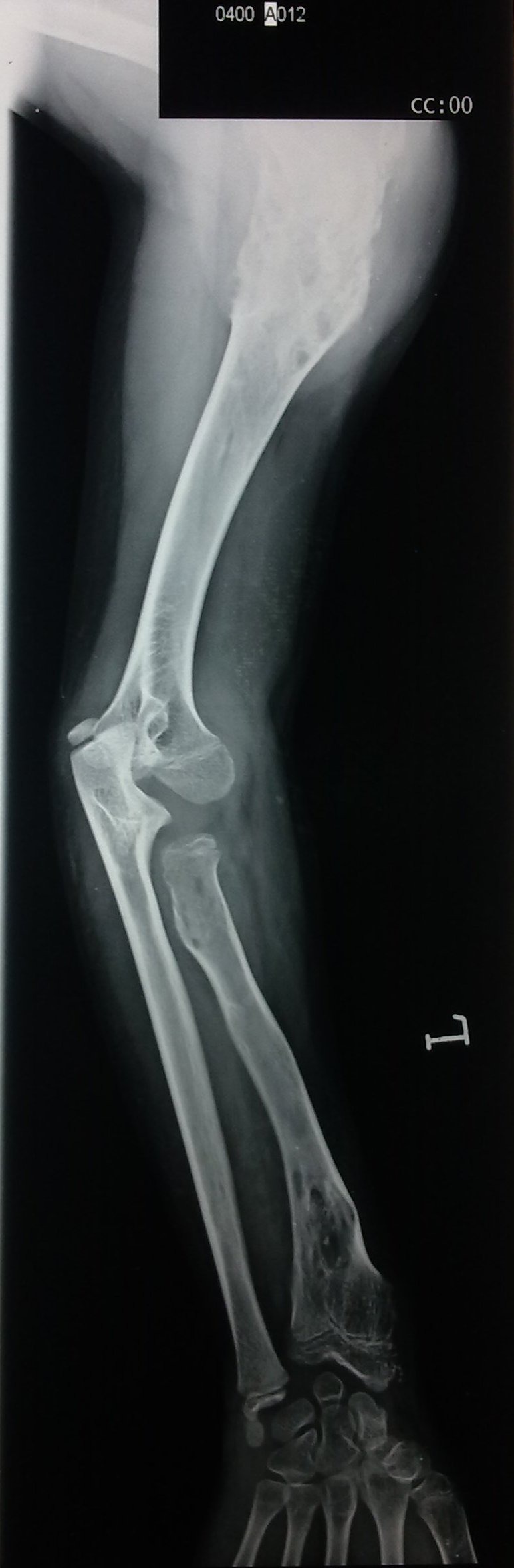
Figure 4: Plain radiograph - left upper limb: enchondromas involving proximal humerus and distal radius.

**Figure:**
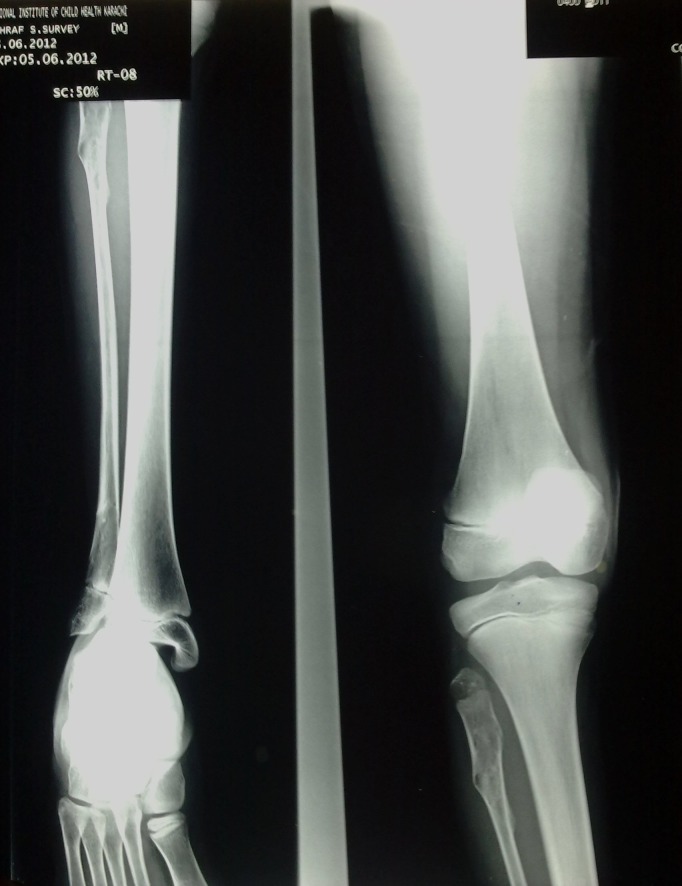
Figure 5: Plain radiograph – left lower limb: enchondromas involving proximal and distal fibula.

Ultrasound of the swelling - right hand revealed a heterogeneous area with blood flow noticed on doppler study. CT angiography revealed large lobulated soft tissue density area, 4.9 x 4.7 cm, showing specks of calcification, representing phleboliths, noted between 2nd and 3rd metacarpal bones. There were no definite bony erosions. Findings were suggestive of hemangioma – right hand. Angiography of right subclavian, axillary, brachial, ulnar and radial arteries was normal. Findings of MRI – right wrist and hand were consistent with a benign vascular lesion most likely haemangioma. Skeletal scintigraphy done with Tc-99m labeled MDP showed increased tracer uptake over mal-aligned upper 3rd of left humerus. An expansile area of increased tracer concentration was also visualized over lower 3rd of left radius and an impression of multifocal bone pathology congruent with radiographic findings was made. Also increased tracer uptake was visualized at 2nd and 3rd metacarpals of right hand, suggestive of involvement of the bones by overlying haemangioma. Rest of the bone scintigraphy findings was normal with bilateral symmetrical tracer uptake in the axial and appendicular skeleton. Histopathology of the swelling right hand confirmed spindle cell hemangioma. On ultrasound abdomen, enlarged spleen (14.2 cm) with no evidence of focal mass or generalized infiltration found.

Parents were counseled about the nature and course of the disease and stressed for regular follow-ups and symptomatic treatment was offered. Orthopaedic consultation was taken for lesions at left upper humerus and distal radius, biopsy was not advised as the patient was asymptomatic. The patient is still in contact with us and orthopaedic team.

## DISCUSSION

Maffucci syndrome (MS) is a rare disorder clinically characterized by childhood onset of enchondromatosis and haemangiomatosis. Exact etiology is still not known however, somatic mosaic mutations in isocitrate dehydrogenase (IDH) 1 and 2 genes have been identified [6]. Some consider it as a variant of Ollier’s disease which has only enchondromatosis [7]. Both genders are equally affected. 

The enchondromas in MS may involve any bone; long bones of the arms and legs and, phalanges being most frequently affected [3]. Bony involvement is asymmetrical and bilateral. Hands were spared by enchondromas in our patient which is in contrary to that reported in the literature. The bony changes in the right hand were due to the pressure effects of the overlying haemangioma. 

The vascular malformations mostly associated with MS are cavernous or spindle cell haemangiomas, later being the most frequent. However, lymphangiomas, arterio-venous malformations may also occur [8]. Haemangiomas involving lip, oropharynx, intra-abdominal and gastro-intestinal tract have also been reported [3, 5, 8]. Thrombi may form, calcify and give a characteristic appearance of phleboliths on plain x-rays [3]. Comparable characteristics were found in our patient who had classical radiological findings on plain x-ray and, histology proven spindle cell haemangioma. But ‘haemangiomatosis’ was not noticed in our patient. This provokes the idea that it is not a classical MS; rather it could be a variant. 

Maffucci’s Syndrome is associated with an increased risk of malignancy especially in enchondromas, transforming to chondrosarcomas. In a recent study by Verdegaal et al [9], overall incidence of developing chondrosarcoma is 40% and patients with enchondromas located in long bones and axial skeleton, especially pelvis, are at more risk. Vascular sarcomas (hemangiosarcoma, lymphangiosarcoma) have also been reported [3]. Beside malignancies in skeletal and vascular components, other malignancies may also occur and the overall frequency of malignancies related with MS is 23% to 100% in different studies [3, 10].

Management is aimed at symptomatic treatment and long-term follow up. Increase in size and pain in the skeletal or soft tissue lesion should raise the suspicion of malignancy and biopsy should be done for exclusion. Corrective osteotomies and limb lengthening procedures for skeletal lesions, and sclerotherapy, irradiation, and surgery for the vascular lesions has been described [10]. A close collaboration is required between the attending physician, paediatric/ orthopaedic surgeon, radiologist and a pathologist.

## Footnotes

**Source of Support:** Nil

**Conflict of Interest:** None declared

